# A structural model of university faculty perceived physical literacy: evidence-based quality physical education

**DOI:** 10.3389/fspor.2025.1691384

**Published:** 2025-10-14

**Authors:** Jiangping Fu, Yi Yu, Bing Cao, Tingting Qiu

**Affiliations:** ^1^ Nantong Institute of Technology, Nantong, Jiangsu, China; ^2^ Wuxi Normal College, Wuxi, Jiangsu, China; ^3^ Zhengde Polytechnic, Nanjing, Jiangsu, China; ^4^ Nanjing Vocational Institute of Railway Technology, Nanjing, Jiangsu, China

**Keywords:** perceived physical literacy, structural mode, quality physical education, faculty members, non-linear education, health ecology

## Abstract

**Background:**

In 2024, China will become an education powerhouse, with requirements for quality sports campus culture.

**Purpose:**

It was used the Perceived Physical Literacy Instrument (PPLI) to explore the factor structure of perceived physical literacy (PL) among university faculties, and to construct a structural model.

**Method:**

It was selected and validated the content validity and consistency of 18 items/versions of the PPLI. Use a snowball sampling method, it is to conduct a questionnaire survey and select university faculty members as research subjects. Using SPSSAU as a computational tool, exploratory factor analysis was first performed, followed by confirmatory factor analysis to determine the factor structure of the structural model.

**Results:**

The content validity and consistency of the PPLI were satisfactory, with an item level 0.83–1 and a scale level 0.94. The structural model was determined to be a 4-factor 12-item factor structure, and the validity was satisfactory. In the exploratory factor analysis, all item loadings ranged from 0.41 to 0.64 (Cronbach's alpha, 0.81–0.87). In the process of calculating the confirmatory factors, all factor loadings ranged from 0.41 to 0.69.

**Conclusion:**

PPLI is an effective and reliable assessment tool for evaluating university faculty members perceived physical literacy (PL perception). A quality sports campus culture should take into account the effectiveness of faculty members faculty PL understanding.

## 1 Introduction

Physical literacy (PL) is a holistic concept as an effective method and means of addressing public health issues (1). In 2007, Whitehead further refined the concept of PL (2). PL is essentially the motivation, confidence, physical ability, knowledge, and understanding that enable individuals to maintain an appropriate level of physical activity (PA) throughout their lifetime (3). There is evidence that PL is central to quality education (4) and is effective for young children and adolescents (5). In 2019, China's strategy to become a sports powerhouse was proposed, and PL was identified as one of the indicators (6). PL may help address the World Health Organization's PA guidelines (7), to address the sedentary behavior and lack of PA among 27.5% of adults, and 81% of adolescents (aged 11–17) worldwide (8).

In 2024, China's strategy to become an education powerhouse was proposed, along with the demand for high-quality sports culture (9). An embedded PL approach (10), a persistent positive adaptation supporting the PL journey of the subsystem life cycle (11), claims that the design of the environment/sports campus culture should be prioritized (12, 13). Contemporary public health/physical activity policy promotion is global and multi-sectoral (14), and faculty members involved in sports campus culture are the primary workers. Literature indicates that the relationship between faculty members' sense of community (environment) and physical education (PL) (15) can form a non-linear educational ecological environment (16–19), which is key to public health/public service (education and physical education) policy implementation. Community consciousness theory suggests that shared interests or activities (20, 21) are the main factors contributing to the outcome of sports culture (22, 23), depending on the management and structure of the program and participants (24–26). Faculty members, particularly those who are non-PE teachers, may play a crucial role in shaping high-quality physical education culture through their sense of community (environment) and connection to PL (9, 11–13). For greater clarity, faculty members (non-physical education teachers) are an important group in fostering a high-quality physical education culture (22–26), and should receive attention.

PL originated from physical education (27) and is based on phenomenological philosophy (28), repeatedly demonstrating that everyone has a unique and specific understanding of the world (28, 29). Exploring PL in an open and information-rich environment may be a reliable and effective measure of PL (30, 31). The Perceived Physical Literacy Instrument (PPLI) (32–34) may be an effective tool for exploring PL in sports campus culture/environments. The PPLI has been found to be reliable in various cross-cultural studies, such as in France (35), Spain (36), Turkey (37–39), China (32–34), and South Korea (39). PPLI has been repeatedly proven to be effective for physical education teachers (40, 41), but none of the authors collected data on faculty (non-physical education teachers) members （FNPETM).

In health ecology, faculty members have both individual (micro-level) attributes (42) and group attributes (intermediate system, connecting sports culture inside and outside the campus) (43). Continuing professional development (CPD) for teachers is considered by UNESCO to be a priority for every country in developing quality physical education (QPE) (40, 44). However, the roles of subject teachers (physical education teachers) and FNPETM in QPE are different (40). Based on perception behavior theory (41, 45), their perceptions and motivations toward PL may also differ, as may the results and impact of future QPE. The motivation for FNPETM (perceived PL) is the basis of the health ecology model (40–45).

Based on the collection and organization of the above data, our study proposes the following objectives: H0, select PPLI to assess the psychological structural characteristics of FNPETM's PL; H1, observe whether FNPETM's PL structural model has the characteristics of a community awareness of non-linear education/campus sports culture (common interests of PL); H2, whether the motivation of FNPETM's PL has the characteristics of QPE's health ecology model.

## 2 Method

### 2.1 Study design

Our study is an assessment of the psychological structural characteristics of perceived PL in university FNPETM, and is a physiological cross-sectional evidence-based study. Prior to the start of the study, the second author applied for ethical review (ZDPC-2024PE-081) from his workplace and obtained permission.

Our research was divided into three phases. The first phase, the research preparation phase, will focus on preparing the PPLI (October–December 2024). The second phase, the research practice phase, will mainly involve distributing questionnaires and collecting data (January–March 2025). The third phase, the research data calculation phase, will mainly involve data calculation and analysis (April–July 2025).

### 2.2 PPLI

PPLI is a fundamental tool in our research process. The 18-item version of the PPLI was selected.

The third author invited two linguists (English teachers, associate professors) to conduct two rounds of English-Chinese translation of the English version of the PPLI to improve the scientific accuracy of the questionnaire.

All authors participated in the preparation of the PPLI. After completing the English-Chinese translation, the basic information section (demographic variables) of the PPLI was finalized. Our study is not intended to be a study of demographic variables. The basic information section has been added for the purpose of identifying respondents. The demographic variables in the PPLI include gender, age, education level, school, year of service, job title, and position.

### 2.3 Sample and data collection

The PPLI was created using the Questionnaire Star online platform as a tool. During the PPLI production process, we set up a method for signing informed consent forms. In the preface section of the PPLI, we informed all respondents that once they began filling out the questionnaire, it would be deemed that they had agreed to and signed the informed consent form.

In order to improve the efficiency of PPLI collection, we have made all items mandatory. Once each respondent completes the questionnaire, data will be generated and stored on the tool platform. The fourth author was responsible for creating the questionnaire and collecting and organizing the data. After the questionnaire is created on the tool platform, it is saved as an electronic QR code for easy distribution. All authors participated in the first round of questionnaire distribution, using WeChat to distribute electronic QR codes for the PPLI at their respective schools.

All respondents were informed that the questionnaire was anonymous and that no personal privacy data would be collected. The data collected was only used for scientific research, and the research was approved by the Medical Ethics Committee. After completing the questionnaire, all respondents were invited to continue sharing the electronic QR code using a snowball method. The fourth author used a tool platform to check whether the sample size met the minimum requirements for the study.

The minimum sample size requirement, based on the 5–20 times requirement for items (46), is set at a minimum of 10 times; taking into account the 1:1 requirement for data calculation (exploratory factor analysis EFA and confirmatory factor analysis CFA) (47), the minimum sample size for the 18-item PPLI is 360 (360 = 180 × 10 × 2).

The PPLI has good reliability and has been repeatedly validated as robust, such as the results of Gendreau J's team (35), Cronbach's *α* > 0.07; rw > 0.7; ICC > 0.7. However, our study was designed to ensure reliability, and the questionnaire was distributed three times.

Our research builds upon the validation of the PPLI for Hong Kong physical education teachers conducted by the SUM team (32–34). The SUM team identified items 4, 5, and 17 as belonging to the first dimension (knowledge and understanding), items 11, 12, and 13 to the second dimension (self-expression and communication with others), and items 2, 7, and 8 to the third dimension (self-perception and self-confidence) (32–34). The psychological structure characteristics of PL among faculty members (non-physical education teachers) may differ from those of Hong Kong PE teachers. This serves as the theoretical basis for conducting CFA calculations of the PPLI in our study (through cross-cultural research methods), specifically for assessing conceptual redundancy and correlation comparisons.

### 2.4 Data calculation

The SPSSAU online platform was selected as the tool for data calculation (48). The data calculation process was divided into three steps.

The first step was to clean the data. Based on data verification standards (FNPETM), data is checked. Then, based on the Grubbs test statistical method (*p* < 0.05, acceptable), each variable was checked for outliers and deleted (47). The research team established the criteria and sequence for eligibility screening: (1) length of service and age, (2) age and degree, (3) duplicate values (incidental responses), (4) Grubbs test (outliers).

We tested the content validity of the data. We invited six experts from the first working group, used a 4-point Likert scale, and then used EXCEL as a calculation tool to complete the assessment of the content relevance of each item in the PPLI. Content validity index (CVI) is first assessed at the item level (I-CVI, ≥0.8, which is an acceptable standard), then at the scale level (S-CVI, ≥0.9, which is an acceptable standard) (49). For demographic variables, we calculated percentages and frequencies. We considered the accuracy requirements of the data, and all data were rounded to the nearest percentage point (unless otherwise specified, such as *P* < 0.001).

The second step is EFA. For SHEETA, EFA was performed using principal component analysis with maximum variance rotation to study the factor structure of FNPETM's PPLI. The calculation standards included in the EFA calculation process are the Kayser-Meyer-Okin (KMO>0.60, statistically significant, sample adequacy verified) value, and the Bartlett test (*p* ≤ 0.001, analyzing the correlation between scale items, sample multivariate normality verified) (50). The number of factors was confirmed by including only items with factor loadings >0.40 and uniqueness values <0.60 (50).

The third step is CFA. Perform CFA on the second subset SHEETB, it was calculated and confirmed the factor structure of the structural model of FNPETM derived from the analysis using cross-validation. The CFA evaluation process includes three indices: absolute fit, parsimonious fit, and incremental fit of the model. We included the following criteria: relative chi-square (X2/ddl; <0.05), root mean square error of approximation (RMSEA; <0.06), adjusted goodness-of-fit index (AGFI; >0.90), non-constrained goodness-of-fit index (NNFI; >0.90), comparative goodness-of-fit index (CFI; >0.90), Tucker–Lewis Index (TLI; >0.90), and Parsimony Goodness-of-Fit Index (PGFI; ≥0.50) (51–55). To further examine the internal consistency of the model, we included Cronbach's α (α ≥ 0.70, relatively high) and Omega (McDonald, *ω* ≥ 0.70, indicating acceptable reliability) (50, 52).

## 3 Results

### 3.1 Sample characteristics

Starting in January 2025, after three data collection rounds, a total of 442 valid data sets were collected.

After data collection was completed, researchers conducted eligibility reviews based on basic information from FNPETM. (1) There may be a logical error in the combination of years of service and age. 4 questionnaires were deleted on the grounds that the data may be true but clearly inconsistent with reality. (2) There may be logical errors in age and degree. Five questionnaires were deleted on the grounds that the data may be true, but there were obvious instances of random filling in. (3) Duplicate numerical records were deleted from 3 questionnaires due to logical inconsistencies in the responses. (4) Grubbs test, 6 questionnaires were deleted due to outliers (47).

Fortunately, all items in the questionnaire were set as mandatory fields during the questionnaire creation process, so there were no missing data in the collected data. After calculation, 424 valid data sets were retained, meeting the minimum sample data requirement of 360 data sets, and data analysis could be performed.

Reliability of retesting: The overall content validity results of the sample are acceptable, with the I-CVI of FNPETM's PPLI ranging from 0.83 to 1.00 (≥0.8) and the S-CVI at 0.94 (≥0.9) (49). By estimating missing values in the dataset, the Cronbach's alpha coefficient was 0.97 (>0.7), indicating acceptable internal consistency (50, 52).

#### 3.1.1 Demographic characteristics

Our study evaluated the psychological structural characteristics of perceived PL of FNPETM (without intention to conduct a control study or sequential study), but we conducted a demographic characteristics analysis and eligibility review, and found that the data met the basic requirements of this study (Table 1).

**Table 1:** Demographic characteristics of the faculty and staff.

Index	Total	Percentage (%)
Gender		
Female	206	48.58
Male^a^	218	51.42
Age		
22–29	158	37.26
30–39^a^	169	39.86
40 above	97	22.88
Education level		
Undergraduate or below^a^	200	47.17
Master	174	41.04
Doctorate	50	11.79
School		
Vocational college	211	49.76
Undergraduate college^a^	213	50.24
Years of service		
1–3 years^a^	199	46.93
4–10 years	130	30.66
11 years or more	95	22.41
Position		
Student affairs department	141	33.25
Teaching department (non-PE teacher)	139	32.78
Supervision department^a^	144	33.96
Job title		
Administrator	161	37.97
Supervisor^a^	182	42.92
Senior supervisor	81	19.11

^a^ Predominating; Total, *N* = 424.

(1) Gender: 218 males (51.42%), and 206 females (48.58%), with fewer females than males. (2) Age: 22–29 years old 158 (37.26%), 30–39 years old 169 (39.86%), 40 years old and above 97 (22.88%), with the 30–39 age group being the most numerous. (3) Educational background: 200 respondents (47.17%) had a bachelor's degree or below, 174 respondents (41.04%) had a master's degree, and 50 respondents (11.79%) had a doctorate degree. The majority of respondents had a bachelor's degree, which is consistent with the educational situation in Chinese universities. (4) Schools: 49.76% of respondents attended vocational colleges (211), and 50.24% attended undergraduate colleges (213); the number of respondents from both types of schools was roughly equal. (5) Years of service: 1–3 years 199 accounting for 46.93%, 4–10 years 130 accounting for 30.66%, 11 years and above 95 accounting for 22.41%, with 1–3 years being the majority (consistent with educational background). (6) Departments: 141 academic management department (33.25%), 139 teaching department (non-PE teachers), and 144 supervision department (33.96%), with the supervision department having slightly more members. (7) Positions: 161 administrators (37.97%), 182 supervisors (32.92%), and 81 senior supervisors (19.11%), with supervisors being the most common (consistent with the educational situation in China).

#### 3.1.2 Data subsets

After demographic characteristics were determined, 424 data sets were divided into two subsets at a ratio of 1:1 (47), using simple random sampling and saved using Excel.

SHEETA212, used for EFA. SHEETB212, used for CFA.

Validity tests were conducted on two separate data subsets. The Cronbach's alpha coefficient for SHEETA was 0.73 (>0.7), indicating acceptable internal consistency; the Cronbach's alpha coefficient for SHEETB was 0.76 (>0.7), also indicating acceptable internal consistency (50, 52).

### 3.2 EFA

Rotate using the maximum variance method and perform principal component analysis (PCA) for EFA. The 18 items of PPLI (*n* = 212) resulted in 12 final items with four factors, as shown in the pattern matrix in Table 2. The structural model with 12 items and 4 factors explained 85.37% of the variance. Of the 18 entries, 12 were observed (items 4, 5, 7, 8, 9, 10, 11, 12, 13, 14, 16, and 17), while 6 were deleted (items 1, 2, 3, 6, 15, and 18).

**Table 2:** Factor structures by exploratory factor analysis and reliability.

Sign	F1	F2	F3	F4	CITC	Communality (h^2^)	Scale alpha
PL04		0.48			0.70	0.71	0.84
PL05			0.45		0.68	0.76	0.81
PL07		0.50			0.67	0.72	
PL08	0.41				0.75	0.83	0.88
PL10		0.51			0.62	0.76	
PL11	0.46				0.68	0.81	
PL12			0.65		0.47	0.54	
PL13	0.52				0.56	0.65	
PL14	0.67				0.48	0.53	
PL17			0.41		0.70	0.78	
PL09				0.47	0.75	0.83	0.87
PL16				0.44	0.72	0.76	
PE	2.15	1.83	1.77	1.48			
%OV	22.68	20.93	20.51	19.31			
C%	24.68	45.61	66.16	85.37			

CITC, corrected item-total correlation; PE, present eigenvalues; %OV, % of variance; C%, cumulative %; F1, the first dimension has the characteristics of knowledge and understanding; F2, the second dimension has physical ability characteristics; F3, the third dimension has the characteristic of confidence; F4, the fourth dimension has motivational characteristics.

The factor loadings for the 12 items ranged from 0.41 to 0.67 (>0.32); the total correlation for the corrected items ranged from 0.53 to 0.83 (>0.4) (50). See Table 3A. This indicates that the factor correlation verification of the model is sufficient. The project content consistency results were satisfactory, with the alpha values of the four factor scales being 0.88, 0.81, 0.84, and 0.87 (>0.7), respectively (50, 52). The KMO index is 0.85 (>0.8), indicating that the validity of the verification sample is basically satisfactory (50). The *p*-value for Bartlett's sphericity test was 0.000 (*p* ≤ 0.001), indicating that the verification sample scale correlation results were suitable for principal component analysis (50).

**Table 3:** Factor structures by confirmatory factor analysis and reliability.

(A) Factor loading coefficient table							
F/D	IN	FL	SE	CR	*P*	R^2^	
F1	PL08	1.00	–	–	–	0.81	
F1	PL14	0.81	66.84	−0.18	0.86		
F1	PL13	0.81	95.84	0.18	0.86		
F1	PL11	0.86	83.53	−0.18	0.86	0.79	
F2	PL04	1.00	–	–	–		
F2	PL10	0.83	0.38	−0.82	0.41	0.78	
F2	PL07	0.91	0.71	−2.13	0.03		
F3	PL05	1.00	–	–	–	0.82	
F3	PL17	0.84	0.34	1.67	0.10		
F3	PL12	0.76	0.39	2.03	0.04		
F4	PL09	1.00	–	–	–		
F4	PL16	0.83	161.37	0.16	0.87		
IN, item number; F/D, factor/domain; FL, factor loading; SE, standard error; CR, critical ratio; P, *p*-value; R^2^, squared multiple correlation; -, Reference items; F1, the first dimension (knowledge and understanding); F2, the second dimension (physical ability); F3, the third dimension (confidence); F4, the fourth dimension (motivational).							
(B) Cross-validation by Confirmatory Factor Analysis and Reliability							
Index	*χ*^2^/df	RMSEA	AGFI	NNFI	CFI	TLI	PGFI
Standards	<3.00	<0.10	>0.90	>0.90	>0.90	>0.90	>0.50
Value	1.03	0.01	0.94	0.94	0.98	0.94	0.53

χ^2^/df, standardized chi-square index; RMSEA, root mean square error of approximation; NFI, non-normed fit index; CFI, comparative fit index; TLI, Tucker–Lewis index; PGFI, parsimonious goodness-of-fit index.

First, delete the 6 cross terms (1, 2, 3, 9, 16, and 18) because they load at 0.40 or higher on two or more factors. However, further PCA analysis of EFA yielded unsatisfactory results, due to the presence of single-item 15. After adding items 9 and 16, four factors (no cross-items) were obtained. An interesting phenomenon is item 15, which again appears as a single item and is therefore ultimately deleted.

### 3.3 CFA

**Figure 1:**
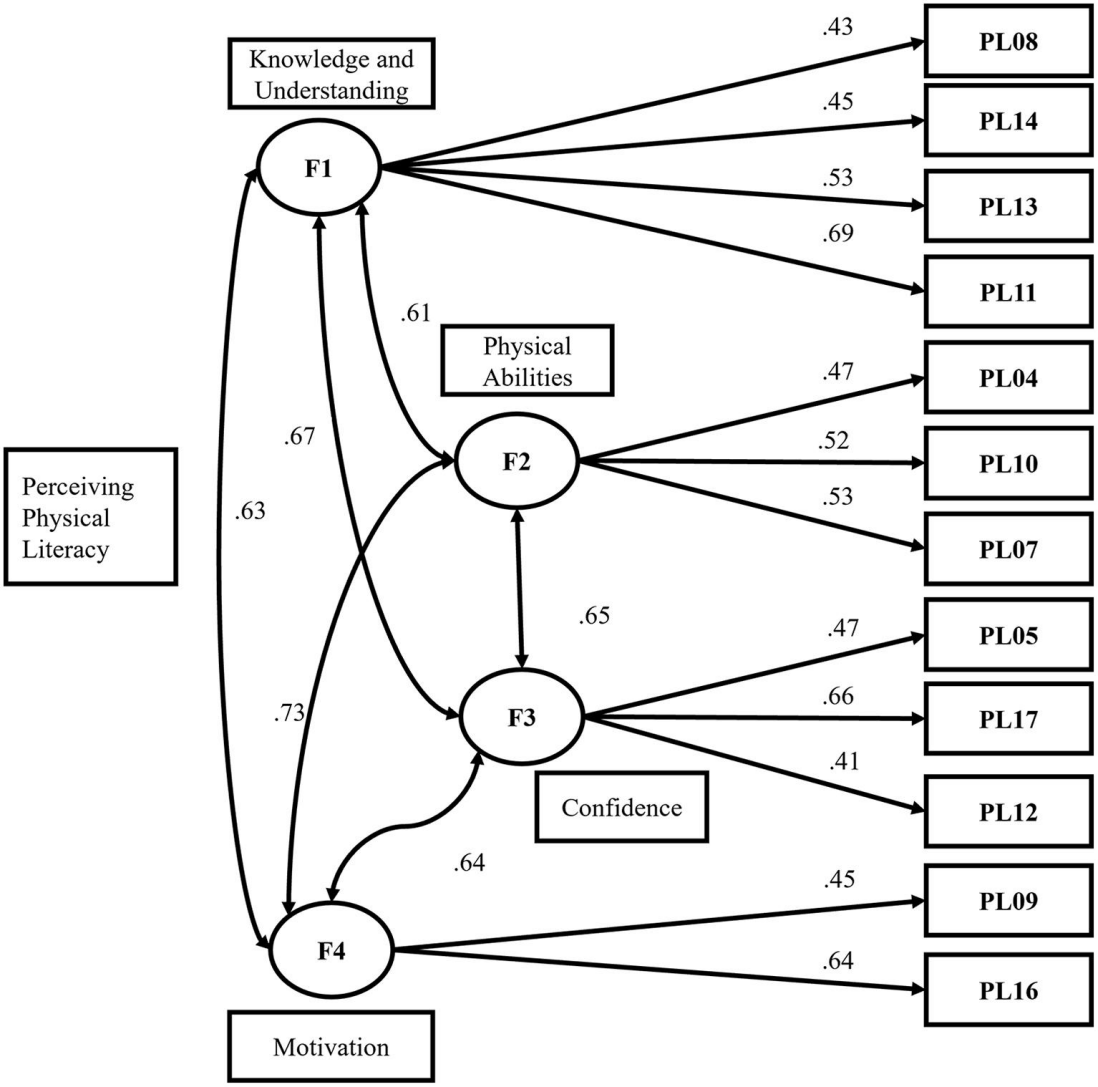
Factor structure and model on university faculty perceived physical literacy.

Cross-validation using CFA retained 12 items, and confirmed the four-dimensional structural model of PPLI for FNPETM (Figure 1).

The structural model of PPLI for faculty members (non-physical education teachers) (Figure 1) comprises: The first dimension (F1, Knowledge and Understanding), including items 8, 11, 13, and 14, with factor loadings ranging from 0.43 to 0.69 (>0.32); The second dimension (F2, Physical Ability) included items 4, 7, and 10, with factor loadings ranging from 0.47 to 0.53 (>0.32); The third dimension (F3, Confidence) included items 5, 12, and 17, with factor loadings ranging from 0.41 to 0.66 (>0.32); The fourth dimension (F4, Motivation), comprising items 9 and 16, with factor loadings ranging from 0.45 to 0.64 (>0.32) (50).

The factor validity results of the CFA were satisfactory, with factor loadings for the 12-item tool ranging from 0.41 to 0.69 (>0.4). The CFA (*n* = 212) structural model had a high degree of fit, with a standardized chi-square index (χ^2^/df) of 1.03 (<3.00). The model's absolute fit indices were RMSEA = 0.01 (<0.10), AGFI = 0.94 (>0.90), and GFI = 0.95 (>0.90). The incremental fit of the model is strong, with NNFI = 0.94, CFI = 0.95, and TLI = 0.94 (all >0.95). The simplicity of the model fit is acceptable, with a PGFI of 0.59 (>0.5). See Table 3B.

## 4 Discussion

Our study used an evidence-based approach to conduct a questionnaire survey on the perceived PL of FNPETM. Through EFA and CFA, a 12-item 4-dimensional structural model was obtained. Based on previous studies conducted by scholars on Chinese physical education teachers and college students, our research is an effective continuation within the scope of campus culture.

### 4.1 Non-linear education

The structural model of FNPETM's PPLI has 12 items and 4 dimensions. The first dimension includes items 8, 11, 13, and 14. According to IPLA and Whitehead (27, 33, 34), the first dimension has the characteristics of knowledge and understanding. The second dimension includes items 4, 7, and 10. According to IPLA and Whitehead (27, 33, 34), the second dimension has physical ability characteristics. The third dimension includes items 5, 12, and 17. According to IPLA and Whitehead (27, 33, 34), the third dimension has the characteristic of confidence. The fourth dimension includes items 9 and 16. According to IPLA and Whitehead (27, 33, 34), the fourth dimension has motivational characteristics.

Compared with the 9-item, 3-dimensional structural model of PPLI for physical education teachers, the psychological structure of perceived PL in FNPETM exhibits significant nonlinear educational characteristics.

The third dimension of the structural model for physical education teachers, consisting of item 4 (core words, positive attitude and psychology), item 5 (core words, appreciation of oneself and others), and item 17 (core words, knowing the benefits), formed a special understanding and comprehension of PA.

Physical education teachers' perception of PL characteristics has formed linear characteristics, because they are the direct executors of daily teaching activities.

The first dimension of the FNPETM structural model, consisting of item 8 (core words, possessing health assessment skills), item 11 (core words, possessing social skills), item 13 (core words, able to handle problems and difficulties), and item 14 (core words, possessing a certain mindset), forms one's perception of knowledge and understanding of PL.

This is the first difference between the non-linear educational characteristics of FNPETM and the linear educational characteristics of physical education teachers. There were also differences between FNPETM's perceived PL/confidence characteristics and physical education teachers' perceived PL/self-awareness and confidence.

The perceived PL/confidence characteristics of FNPETM are the third dimension of the structural model, comprising item 5 (core words, appreciating oneself or others), item 12 (core words, wild/natural survival), and item 17 (core words, knowing that exercise is good for health).

Physical education teachers' perception of PL/self-awareness and confidence is the second dimension of the structural model, including item 11 (core words, social skills), item 12 (core words, wild/natural survival), and item 13 (core words, ability to handle problems and difficulties).

FNPETM's perception of PL/confidence seems to focus more on breadth of scope, while physical education teachers' perception of PL/self-awareness and confidence seems to focus more on method/means orientation (24–26). All researchers believe that this is the second difference between the non-linear educational characteristics of FNPETM and the linear educational characteristics of physical education teachers.

The structural model of FNPETM's perception of PL has motivation (fourth dimension, items 9 and 16), and physical ability (second dimension, items 4, 7, and 10). The structural model of physical education teachers' perception of PL, the first dimension is self-expression and communication with others, including items 2, 7, and 8.

All researchers believe that these two dimensions cannot be directly analyzed for correlation, but if the prerequisites for non-linear education (shared interests and activities in sports campus culture) (16–19) are introduced, the differences between the two can be analyzed indirectly.

The shared interests and activities of physical education teachers and students in campus sports culture are primarily carried out through physical education classes, which provide students with orderly (direct) assistance.

FNPETM shares students' interests and activities in sports campus culture, which is based on sports activities (non-teaching activities) that help students form an orderly (indirect) or atmospheric environment (24–26).

To put it more clearly, there are different requirements for physical education teachers and FNPETM in terms of shared interests and activities in sports campus culture.

In order to stimulate students' interest in learning through sports campus cultural activities, FNPETM may need to make more extensive preparations.

This may be the third difference in perceived PL observed in our study between physical education teachers and FNPETM.

Our study does not intend to evaluate the merits of physical education teachers' and FNPETM's perceived PL, but only to describe the differences in their perceived PL (the phenomenon we have observed so far).

### 4.2 Health ecology model

Health ecology models have micro (individual), miso (group), macro (policy), and social (public health and services) levels (42, 43). University FNPETMs (Sports Campus Cultural Workers, non-sports teachers) are frontline workers in public health and services, implementers of sports campus cultural policies, an important part of the sports campus cultural community, and also bearers of their own health and wellness journeys.

QPE's priority is teachers’ CPD (40, 44), and university FNPETM as sports campus culture workers should be given attention. Researchers conducted an in-depth analysis from the perspective of QPE, focusing on the possibility of universities and college students jointly forming a healthy ecosystem (using the perception of whether PL motivation has common interests and activities, as the standard for forming community awareness).

The structural model of college students’ perception of PL includes 9- items 3- dimensions. College students' motivation dimensions of the PL structural model include item 5 (core words: appreciation of self and others), item 17 (core words: knowing the benefits of exercise), and item 18 (core words: desire to understand trends).

The motivational dimensions of FNPETM's perceived PL structural model include item 9 (core words, exercise improves health) and item 16 (core words, exercise promotes friendship). It can be seen that the motivation for perceiving PL in FNPETM has an intrinsic characteristic, while the motivation for perceiving PL in college students has a conditional characteristic (external information as a trigger).

We can infer that when the intrinsic motivation of FNPETM and the contingent motivation of college students promote common interests and activities that foster a sense of community, it creates an environment/atmosphere conducive to sports campus culture, ultimately forming a healthy ecosystem on college campuses.

It is interesting to note that during the EFA process, items 9 and 16 could not form a valid structural model after being deleted for the first time. At the same time, we also attempted to merge items 9 and 16 into the third dimension of the structural model, to form a structural model similar to that of physical education teachers or college students, but we failed. Ultimately, the motivational dimension of perceived PL in FNPETM was retained separately, serving as strong evidence for FNPETM, which is a CPD of a health ecology model/QPE (40, 44).

### 4.3 Limitations and advantages

#### 4.3.1 Advantage

Our study observed the CPD of QPE in a grand health ecology model using male ambition. We provide strong evidence (indirect) for the formation of community awareness (shared interests and activities) in sports campus culture through the psychological structural characteristics of perception PL at FNPETM University.

#### 4.3.2 Limitations

Although data collection was conducted using a snowball sampling method, it does not represent complete randomness, which being potential biases exist around institutional networks. The comparative analysis of the structural model of PPLI with physical education teachers and college students is based on local meta-analysis logic and does not represent a universal phenomenon.

## 5 Conclusion

PPLI, as a tool for measuring perceived PL, may be appropriate for FNPETM in universities. Our research results prove that the structural model/evaluation of PPLI is reliable and effective. The 18 PPLI entries that were deleted may be cases that were not observed in this study. Further research on the health ecology model and QPE can be conducted using sports campus culture as a covariate.

## Data Availability

The original contributions presented in the study are included in the article/Supplementary Material, further inquiries can be directed to the corresponding author.
